# The June Meeting at Old Point Comfort

**Published:** 1899-10

**Authors:** 


### THE JUNE MEETING AT OLD POINT COMFORT.

The National Dental Association, at its recent meeting at Niagara, adjourned to meet at Old Point Comfort during the last week in June, 1900. This change of time was made so that the annual meeting might not interfere with the prospective visit of many to the International Dental Congress, to be held at Paris during the Exposition. This was the proper course to take, but why confine the change solely to the present year?

It has been long evident to many that the selection of August as the dental convention period has been a mistake. It is the most uncomfortable portion of the year, and is at a time when the tired worker desires and needs absolute rest, mental and physical. The result is that many of the best men in the profession will not take part in the national gatherings, preferring to sacrifice professional interests to physical and mental needs. Those who unselfishly make the sacrifice generally do it at a loss in both directions.

This departure from a time-honored custom may open the door to a permanent change; if so, it will be a valuable experience. The mistake has been made in selecting the last of June. This period is usually the hottest experienced during the year upon the Atlantic coast, while the early part of June is generally the pleasantest period.

The time is none too long to prepare for this gathering. It would be discouraging to have the meeting fall far behind the one held at Niagara, but we have faith in the energy of the president and his co-workers, and do not question but that the national meeting at Old Point Comfort will gather many not often seen at these conventions. To effect this, however, a programme worthy the occasion must be provided.

